# Paternal Obesity Induced by High-Fat Diet Impairs the Metabolic and Reproductive Health of Progeny in Rats

**DOI:** 10.3390/metabo13101098

**Published:** 2023-10-20

**Authors:** Carlos Larqué, Haydée Lugo-Martínez, Xiadany Mendoza, Monserrat Nochebuena, Luis Novo, Ricardo Vilchis, Guadalupe Sánchez-Bringas, Laura Ubaldo, Myrian Velasco, Rene Escalona

**Affiliations:** 1 Laboratory of Embryology and Genetics, Departamento de Embriología y Genética, Facultad de Medicina, Universidad Nacional Autónoma de México, Mexico City 04510, Mexico; skiuty@hotmail.com (C.L.); haydeelugo@umam.mx (H.L.-M.);; 2 Departamento de Anatomía, Facultad de Medicina, Universidad Nacional Autónoma de México, Mexico City 04510, Mexico; 3 Neuroscience Division, Department of Cognitive Neuroscience, Instituto de Fisiología Celular, Universidad Nacional Autónoma de México, Ciudad Universitaria, AP 70-253 Coyoacán, Mexico City 04510, Mexico

**Keywords:** obesity, high-fat diet, reproduction, fertility, metabolism, intergenerational

## Abstract

Due to the increased incidence of obesity, it is of great importance to identify all the possible consequences in those who suffer from it and their descendants. This study aimed to investigate how paternal obesity, resulting from an 18-week high-fat diet (HFD), affects the metabolic and reproductive health of offspring. In the fathers (F0 generation), the HFD led to significant weight gain, primarily due to increased visceral fat. It also resulted in impaired glucose control and reduced insulin sensitivity. Furthermore, F0 males from the HFD group had reduced sperm concentration and lower sperm viability but were still able to sire litters. F1 offspring were monitored during 18 weeks; F1 offspring from obese fathers displayed increased body weight during the experimental window, especially in males, without significant metabolic disturbances. Additionally, F1 males showed reduced sperm viability, indicating potential reproductive implications. On the other hand, F1 females showed normal estrous cycle patterns but had a reduced number of primordial follicles, suggesting a decrease in their follicular reserve and reproductive potential. This study highlights that metabolic and reproductive issues may be passed down to future generations through the paternal line.

## 1. Introduction

Overweight and obesity rates have increased steadily over the past decades [1]. The obesity epidemic represents a severe public health issue since excess body weight impairs multiple physiological processes. Concomitantly, infertility rates have been attracting attention. In 2010, it was reported that infertility affected approximately 48.5 million couples worldwide; however, the precise number might be significantly higher [2]. Infertility is a complex multifactorial disease; nevertheless, it is widely accepted that obesity is one of the most critical risk factors for reduced fertility in females and males [3,4,5,6].

Furthermore, the consequences of obesity are not limited to those who experience it; there is accumulating evidence that obesity might impair the health of the offspring. This concept of the developmental origin of health and disease (DOHaD) states that the exposure of the fetus to an adverse environment (e.g., an abundance or scarcity of nutrients) predisposes the organism to developing diseases during adulthood [7]. In this context, the impact of maternal obesity on the metabolic health of their progeny has received much attention. In humans, maternal diabetes and obesity can impair fetal growth [8]. Furthermore, experiments in female mice fed a high-fat diet (HFD) have shown that their subsequent generation develops a higher body mass and a constellation of metabolic disturbances [9]. As previously stated, given the predominant role of the mother during the perinatal period, maternal obesity has been the central focus of most trans- and intergenerational studies regarding obesity. However, the role of paternal obesity over the health of its offspring has been steadily gaining relevance.

For example, epidemiological studies have shown that increased paternal BMI is linked to increased birth weight and BMI during childhood, independent of maternal BMI or metabolic status [10]. Animal studies have shed further light on this regard; for example, paternal HFD exposure impairs pancreatic beta cell function in their offspring in a sex-dependent manner [11]. Furthermore, obese mice fed an HFD sired male and female progeny (F1) with increased body weight and several metabolic disturbances, even when the F1 generation was only fed standard chow [12].

Given the intricate relationship between metabolic status and reproduction, it seems feasible to assume that parental obesity might impair the reproductive health of their progeny. For example, female rats sired by obese mothers fed a cafeteria diet have been shown to have a precocious vaginal opening, altered follicle counts, and fewer healthy oocytes [13]. On the other hand, male rats sired by mothers fed an HFD, in addition to increased body weight, displayed seminiferous tubules with abnormal morphology, a decreased sperm count, and reduced plasma testosterone [14]. Additionally, the work of Fullston and colleagues has shown that paternal obesity can induce metabolic disturbances in the progeny, as well as reduced sperm motility in their F1 male offspring. In contrast, the female F1 offspring displayed molecular alterations in their oocyte cumulus cell complexes [15,16]. Taken together, these results indicate that paternal obesity plays a vital role in determining the metabolic health of the progeny and might also be involved in determining some aspects of the reproductive potential of subsequent generations. This paper aims to determine alterations in the gametogenesis and other reproductive parameters of male and female rats sired by obese fathers fed an HFD. In male F1 from both groups, sperm production and viability were evaluated. As an exploratory mechanism, we assessed spermatogonial proliferation by proliferating cell nuclear antigen (PCNA) staining, and Wilms tumor protein (WT1) was employed for the labeling and quantification of Sertoli cells; finally, we determined testosterone concentration in plasma on the basis that these elements have been shown to be impaired upon the administration of an HFD [17,18,19]. On the other hand, in F1 females, we analyzed follicular growth and recruitment; furthermore, we performed relative quantification of anti-Müllerian hormone (AMH) in different follicle types as a possible mediator of follicle arrest [20].

## 2. Materials and Methods

### 2.1. Animals and Experimental Design

Five-week-old male Wistar rats were randomly divided between the control group (CTRL, *n* = 6) and the high-fat group (HFD, *n* = 12) to establish the founding generation (F0). Rats were housed in acrylic cages (45 × 30 × 20 cm) in pairs under a controlled temperature (22 ± 2 °C). For 18 weeks, the animals had *ad libitum* access to water and food, according to their experimental group. Bodyweight gain and caloric intake were monitored weekly for both groups. At the end of the experimental period, males from both groups (CTRL *n* = 3; HFD *n* = 5) were mated to young female rats (200 g) to obtain their offspring (F1). At birth, all litters were culled to 8 pups to ensure that all animals had equal access to food. For each litter (representing one F0 male), two male and two female offspring were randomly selected for analysis, amounting to a total of *n* = 10 for F1 _HFD_ and *n* = 6 for F1 _CTRL_, for both male and female F1 offspring. After weaning, the F1 offspring were given free access to control chow and water for 15 weeks. As with the F0 generation, body weight gain and caloric intake were monitored weekly for all groups. The committee for ethical evaluation at the Facultad de Medicina UNAM approved the experiments according to international guidelines for the ethical use of animals. Procedures aimed to minimize the number of animals and their suffering.

### 2.2. Diet

Animals allocated in the CTRL group were fed standard rodent chow (Laboratory Rodent Diet 5001, LabDiet^®^; Minneapolis, MN, USA). The CTRL diet had a caloric output of 3.36 kcal/gram, with 13%, 28%, and 59% calories provided by fats, protein, and carbohydrates, respectively. The HFD diet was manufactured in the Department of Embryology and Genetics of Facultad de Medicina, UNAM. In brief, for every 100 g of HFD, 49.5 g of ground Laboratory Rodent Diet 5001 pellets, 17.5 g of lard, 17.5 g of olive oil, and 15 g of lyophilized egg albumen were thoroughly mixed and refrigerated before being administered to the animals. The HFD diet had a caloric output of 5.41 kcal/gram, with 62%, 20%, and 18% calories provided by fats, protein, and carbohydrates, respectively.

### 2.3. Intraperitoneal Glucose Tolerance Test (IPGTT) and Insulin Tolerance Test (ITT)

One week before the end of the experimental period, the animals were fasted for 12 h to determine fasting plasma glucose (FPG) and to perform the IPGTT and ITT. For IPGTT, 2 g glucose/kg bodyweight was administered intraperitoneally; in the case of ITT, 0.2 IU/kg of human recombinant insulin (Humulin, Lilly; Indianapolis, IN, USA) was administered. Blood glucose was measured at 15, 30, 60, 90, and 120 min after the intervention using a handheld blood glucose monitor (Accu-Chek Instant, Roche; Indianapolis, IN, USA).

### 2.4. Estrus Cycle Analysis

The external genitalia of female offspring (F1) were monitored each morning from weaning to determine vaginal opening. Estrous cycle determination was carried out by vaginal smears, which were obtained for 14 days beginning on postnatal day 60. Vaginal smears were obtained by gently pipetting 100 μL of warm sterile saline solution (0.9% NaCl) into the rat’s vagina. The solution was smeared over gelatin–coated microscope slides and stained with hematoxylin–eosin. The estrous cycle phase was determined according to the predominant cell types in each slide under a microscope [21]. Briefly, proestrus is characterized by an abundance of nucleated cells and few cornified cells (5:1), estrus is characterized by the presence of cornified and nucleated cells (1:1), metestrus is characterized by the abundance of leukocytes and few nucleated cells (10:1), and diestrus is characterized by abundant leukocytes and few nucleated cells (3:1). Rats were considered regular if they exhibited 4–5-day cycles with the correct progression between phases. Thus, rats with longer phases or those who presented an incorrect progression of stages were considered to have irregular cycles.

### 2.5. Sperm Analysis

Sperm were obtained by cutting the cauda of the epididymis, according to previous reports [22]. Briefly, using a pair of scissors and tweezers, the contents of the cauda were released by squeezing in 5 mL of PBS within a clean Petri dish. The suspension was incubated for 15 min at 37 °C and was immediately loaded into a hematocytometer to assess sperm concentration. To assess sperm viability, vital staining was performed. One drop of the sperm suspension was stained with an equal volume of eosin (1%)–nigrosine (10%) solution [23]. Sperm viability was determined by analyzing 200 sperm at random under 40× magnification.

### 2.6. Tissue Extraction

At the end of each experiment, all rats were euthanized after a 12 h fasting period with a pentobarbital overdose (Pisabental, Aranda; 75 mg/kg; Mexico City, Mexico). Blood was extracted by cardiac puncture and was collected in EDTA-containing tubes. Plasma was separated by centrifugation and stored at −80 °C until analyzed. Gonadal tissue was removed and washed in ice-cold phosphate buffer saline (PBS), followed by fixation in 4% paraformaldehyde in PBS at 4 °C. After fixation, the tissues were dehydrated and embedded in paraffin for sectioning.

### 2.7. Histological Analysis

To study histological alterations in the gonads of F1 animals, an analysis of hematoxylin–eosin-stained paraffin sections was performed in all groups. In the case of the testes, for each animal, ten random fields scattered across the 5 μm section were selected for microscopic analysis. In each field, transversal sections of seminiferous tubules were examined. The area of the seminiferous tubules and the thickness of the germinal epithelia were assessed with the aid of ImageJ software. Follicular development was assessed in paraffin sections using one ovary at random from each animal. The ovaries were cut into 5 μm thick sections, every tenth section was collected on glass slides, and a total of 4–5 sections per ovary were analyzed. Follicular stages were classified according to previously reported criteria [24], and the total number of follicles per stage were normalized by unit of area (10,000 μm^2^).

### 2.8. Immunofluorescence Staining

Immunofluorescence was performed on representative paraffin sections of each animal. Sections were deparaffinized and hydrated in a series of xylol–ethanol–water washes. The tissue sections were antigen retrieved in citrate buffer (0.01 M sodium citrate, 0.05% Tween 20, pH 6.0) for PCNA and AMH; meanwhile, Tris/EDTA buffer (0.01 M Tris Base, 0.05% Tween 20, pH 9.0) was used for WT1. The sections were then blocked for 2 h at RT in 2% BSA, 2% NGS, and 0.5% Triton X-100 in PBS; posteriorly, the sections were incubated with the primary antibodies for 24 h at 4 °C. The following primary antibodies were used: mouse anti-AMH (1:100; SC-166752, Santa Cruz, TX, USA); mouse anti-WT1 (1:100; SC-7385, Santa Cruz, TX, USA); and rabbit anti PCNA (1:500; ab92552, Abcam; Cambridge, UK). The slides were washed twice in PBS-0.05% Tween and incubated 2 h at RT with the following secondary antibodies: Alexa Fluor goat anti-mouse 488 (1:800; 115-545-166, Jackson ImmunoResearch; West Grove, PA, USA) and Alexa Fluor goat anti-mouse 647 (1:400; 111-605-003, Jackson ImmunoResearch; West Grove, PA, USA). Then, the slides were washed twice in PBS-0.05% Tween, incubated for 10 min at RT with DAPI (1 μg/mL; 28718-90-3, Santa Cruz, TX, USA), and mounted in MOWIOL 4-88 solution (475904, Merck Millipore; Hesse, Germany). Fluorescent images were captured using a confocal microscope (LSM 880 Zeiss; Oberkochen, Germany), laser intensity and digital gain were held constant between all groups. Images were analyzed using ImageJ software to measure mean fluorescence intensity and the number of positive cells. For males, 10 random seminiferous tubules were analyzed per animal. In the case of females, 3 representative follicles of each category were analyzed per animal.

### 2.9. Testosterone Determination

Plasma testosterone was determined using the Milliplex^®^ MAP Multi-Species Hormone Magnetic Bead Panel (MSHMAG-21K; Merck Millipore; Hesse, Germany), according to the manufacturer’s instructions.

### 2.10. Statistical Analysis

Origin2019 software was used for statistical analysis; data are presented as means ± standard error of the mean (SEM). A two-tailed unpaired Student t-test was used for two-group comparisons. Sample sizes are indicated within the figure legends, with *p* < 0.05 considered statistically significant.

## 3. Results

### 3.1. HFD Induces Obesity and Impaired Glucose Homeostasis in F0 Males

**Figure 1:**
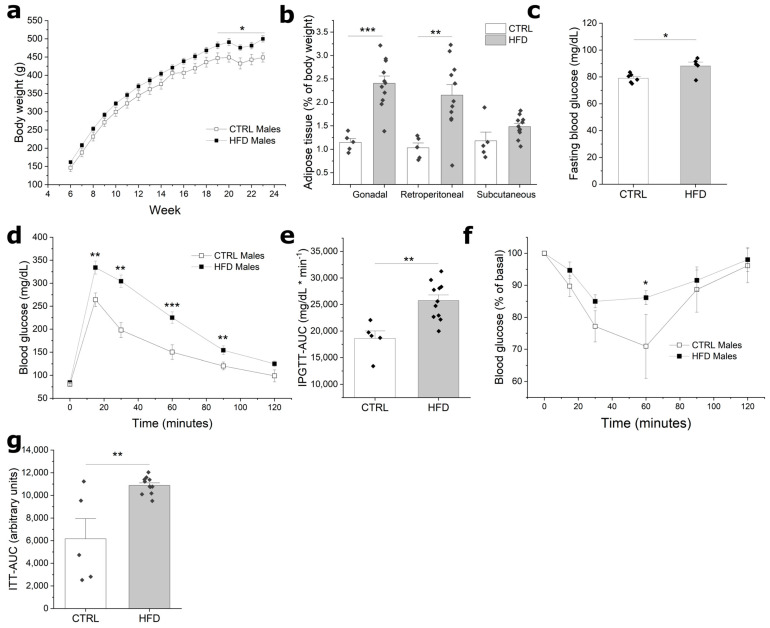
Somatometric and metabolic characterization of F0 males after 18 weeks of diet administration. (**a**) Body weight gain of CTRL and HFD males during 18 weeks of the experimental period; (**b**) weight of adipose tissue pads normalized to the weight of the animals; (**c**) fasting blood glucose; (**d**) intraperitoneal glucose tolerance test of CTRL and HFD rats; (**e**) area under the curve for the intraperitoneal glucose tolerance test; (**f**) insulin tolerance tests of CTRL and HFD rats; (**g**) area under the curve for the insulin tolerance test of CTRL and HFD rats. Bars represent mean ± SEM; *, **, and *** scripts represent *p* ≤ 0.05, ≤0.01, and ≤0.001, respectively. *n* = 12 and 6 for HFD and CTRL, respectively.

To examine the effect of paternal obesity on the metabolic and reproductive health of rat progeny, we exposed five-week-old male Wistar rats to an HFD for 18 weeks. In F0 males, the HFD treatment induced a significant weight gain beginning at week 15 (Figure 1a). By week 18, the HFD group displayed a body weight 11.4% superior to that of the CTRL group. *Post-mortem* analysis revealed that this weight difference was mainly due to an expansion of visceral adipose tissue depots. Gonadal and retroperitoneal adipose tissue depots were significantly heavier in the HFD group compared to the CTRL group (Figure 1b). These adipose tissue pads were approximately twice as heavy in the HFD group when adjusted to the total mass of the animal. On the other hand, subcutaneous fat did not show a significant difference between groups. In addition to increased fat mass, the HFD induced impaired glucose homeostasis (Figure 1c–e). Animals from the HFD group displayed an increased fasting plasma glucose relative to the CTRL group (Figure 1c; 88.5 and 78.6 mg/dL, respectively; *p* < 0.05). Additionally, the HFD group exhibited alterations in their intraperitoneal glucose tolerance test (Figure 1d,e). Furthermore, in the intraperitoneal insulin tolerance test, the HFD group displayed reduced insulin sensitivity (Figure 1f,g). Thus, HFD treatment induced paternal obesity and impaired glucose homeostasis and insulin-mediated glucose uptake.

### 3.2. Paternal Obesity Reduces Reproductive Potential

In order to establish the F1 generation and determine the reproductive disturbances caused by obesity, at the end of the experimental period, F0 males were mated to young female Wistar rats. Despite having relatively smaller testes, all males were capable of generating a litter; moreover, all litters were on average of a similar size (Table 1). Additionally, testes were processed for HE staining, and microscopic analysis revealed that there were no differences between groups in the morphology of seminiferous tubules. However, during sperm analysis, it was found that the HFD group had a significant reduction in sperm concentration and in the proportion of live sperm (Table 1). Additionally, HFD-fed males displayed a tendency towards reduced plasma testosterone. Hence, although there were no significant changes in fertility, litter size, and testis morphology, HFD impaired sperm production in HFD-fed F0 males.

### 3.3. F1 from HFD Fathers Have Increased Body Weight without Metabolic Alterations

**Figure 2:**
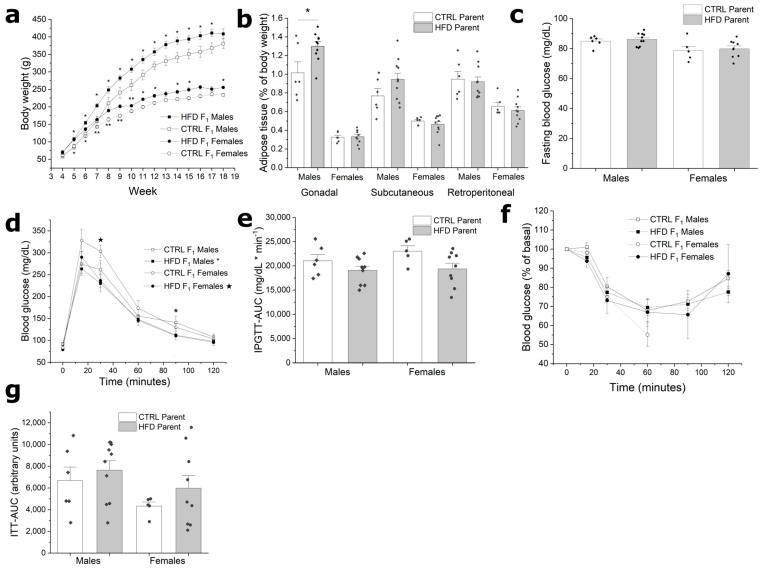
Somatometric and metabolic characterization of 18-week-old F1 _CTRL_ and F1 _HFD_ offspring. (**a**) Body weight gain of F1 males and females during the experimental period; (**b**) weight of adipose tissue pads normalized to the weight of the animals; (**c**) fasting blood glucose; (**d**) intraperitoneal glucose tolerance test; (**e**) area under the curve for the intraperitoneal glucose tolerance test; (**f**) insulin tolerance test; (**g**) area under the curve for the insulin tolerance test. Bars represent mean ± SEM; * script represents *p* ≤ 0.05 compared to F1 _CTRL_. *n* = 10 and 6 for F1 _HFD_ and F1 _CTRL_, respectively.

Paternal obesity increased the body weight of offspring from the fourth week of life into early adulthood (Figure 2a). By 10 weeks of life, F1 from HFD males were significantly heavier compared to F1 from the CTRL males (males 17%, *p* = 0.02; females 8.1%, *p* = 0.01). However, in subsequent weeks, this weight difference became less evident. Despite increased body weight, body composition in the F1 _HFD_ group was not significantly increased compared to their respective controls (Figure 2b). However, only F1 _HFD_ males displayed an increase in the proportion of gonadal fat. On the other hand, while the F1 _HFD_ group was generally heavier than the F1 _CTRL_ group, they did not develop any apparent metabolic disturbances. Fasting plasma glucose was similar between the F1 _HFD_ and F1 _CTRL_ groups (Figure 2c). In the case of the intraperitoneal glucose tolerance test, F1 _HFD_ individuals showed a tendency for a reduced curve (Figure 2d). However, there were no significant differences when comparing the area under the curve (Figure 2e). Furthermore, during the insulin tolerance tests, there were no differences between the F1 _HFD_ and F1 _CTRL_ groups (Figure 2f,g). However, it is worth mentioning that in the insulin tolerance test, all F1 _CTRL_ females had to be removed from the experiment at minute 60 due to severe hypoglycemia. On the contrary, only 50% of F1 _HFD_ females had to be removed from the experiment. In summary, while the F1 _HFD_ group had an increased body weight, there were no significant changes in adiposity; additionally, there were no associated metabolic alterations.

### 3.4. Paternal Obesity Impairs Sperm Production in F1 Males

**Figure 3:**
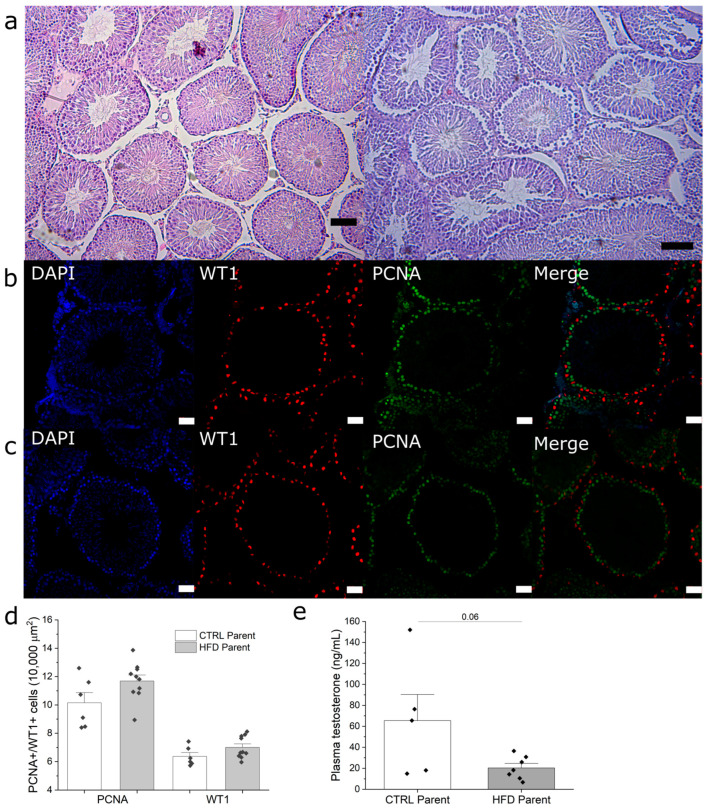
Morphologic analysis of testes from 18-week-old F1 males. (**a**) HE-stained paraffin sections of F1 _CTRL_ (left) and F1 _HFD_ (right) testes; (**b**,**c**) immunofluorescence staining for WT1 (red) and PCNA (green) in testes from F1 _CTRL_ and F1 _HFD_ males, respectively; (**d**) quantification of WT1+ and PCNA+ cells per area unit in F1 _CTRL_ and F1 _HFD_ testes; (**e**) plasma testosterone in F1 males from both groups. Bars represent mean ± SEM. In panel d, *n* = 10 and 6 for F1 _HFD_ and F1 _CTRL_, respectively. In panel e, *n* = 7 and 5 for F1 _HFD_ and F1 _CTRL_, respectively. Black and white bars represent 200 μm and 20 μm, respectively.

F1 _HFD_ males had no differences in testis weight compared to F1 _CTRL_ males (Table 2); furthermore, there were no significant differences in total sperm count between the groups (Table 2). However, when sperm viability was analyzed using the eosin–nigrosine stain, the F1 _HFD_ group showed a reduction in viable sperm (Table 2). Given this reduction, we investigated if there were any morphological alterations in seminiferous tubules as an indicator of impaired gametogenesis. Both seminiferous tubule area and gross morphology were similar between groups (Table 2; Figure 3a). When testis sections were analyzed for the expression of the proliferation cell marker PCNA, there were no significant differences between groups (Figure 3b–d). Finally, similar results were obtained for WT1 staining, a Sertoli cell marker (Figure 3b–d). Furthermore, F1 _HFD_ showed a trend in decreased plasma testosterone (Figure 3e, *p* = 0.06). Taken together, these results indicate that while F1 _HFD_ had lower sperm viability, there were no evident alterations in the seminiferous tubules, as evidenced by HE, WT1, and PCNA staining.

### 3.5. Paternal Obesity Reduces Follicular Reserve in F1 Females

**Figure 4:**
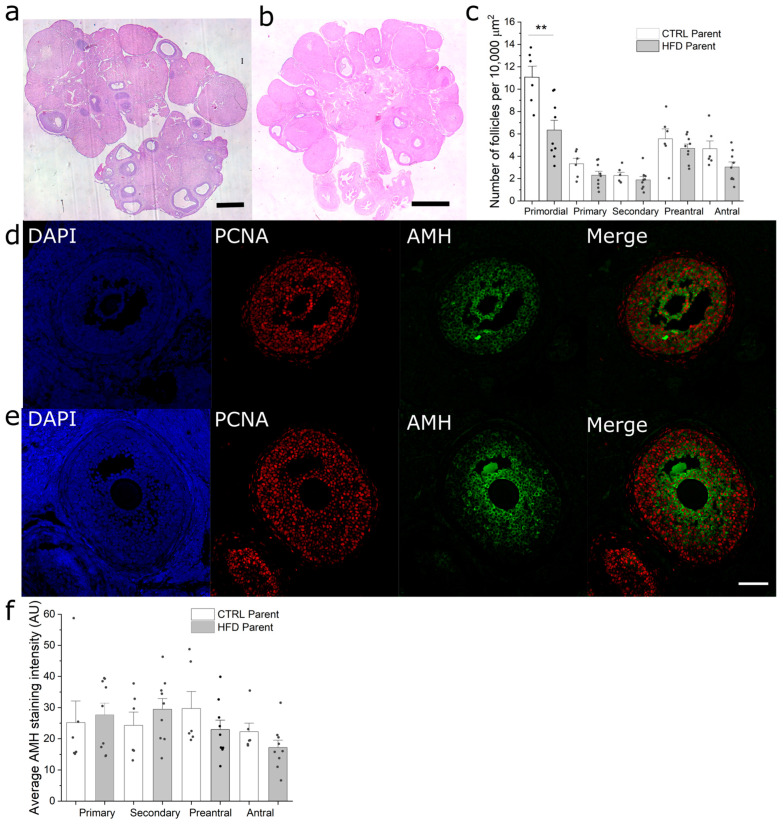
Morphologic analysis of ovaries from 18-week-old F1 females. (**a**,**b**) HE-stained paraffin sections of F1 _CTRL_ and F1 _HFD_ ovaries, respectively; (**c**) quantification of the number of follicles per area unit in ovaries from F1 _CTRL_ and F1 _HFD_ females; (**d**,**e**) immunofluorescence staining for AMH (green) and PCNA (red) in ovaries from F1 _CTRL_ and F1 _HFD_ females, respectively; (**f**) quantification of AMH staining intensity in follicles from F1 _CTRL_ and F1 _HFD_ females. Bars represent mean ± SEM; ** script represents *p* ≤ 0.01 compared to F1 _CTRL_. *n* = 9 and 6 for F1 _HFD_ and F1 _CTRL_, respectively. Black and white bars represent 0.5 mm and 50μm, respectively.

F1 females were monitored daily to determine the onset of vaginal opening as a marker of sexual maturation; however, there were no differences between groups. On the other hand, beginning on postnatal day 60, all F1 females were subjected to daily vaginal smears to assess their estrous cycle for two weeks. Both estrous cycle duration and phase progression were similar between both groups. Furthermore, to determine if paternal obesity affected follicular reserve and development, we analyzed HE-stained paraffin sections of the ovaries. Although both groups displayed similar morphologies (Figure 4a,b), F1 _HFD_ females showed fewer primordial follicles in their ovaries compared to F1 _CTRL_ females (Figure 4c). However, when we compared the remaining categories of follicles between groups, no differences were observed. Finally, to further assess follicular development, the relative expression of AMH was determined in the follicles of both groups (Figure 4d–f). No differences were observed in any of the categories of follicles analyzed. Thus, paternal obesity reduced follicular reserve, as indicated by the reduced number of primordial follicles; however, it did not significantly impair follicular recruitment and growth.

## 4. Discussion

Given the widespread increase in obesity and overweight incidence across the globe, it is of great importance to investigate the reach of this problem. The detrimental role of maternal obesity over progeny growth and development in utero has been extensively accepted. However, recent evidence has put forward the importance of paternal obesity in dictating the metabolic and reproductive health of progeny. This paper shows that paternal obesity in rats can induce accelerated body weight gain in their male and female progeny. Furthermore, this is one of the few studies to evaluate the sexual dimorphism of the reproductive impairment caused by paternal obesity. Epidemiological data have shown that paternal obesity can impair development, beginning in the blastocyst stage in humans [25]. Furthermore, these effects are not limited to the gestational period, but are also present in the postnatal stage. For example, increased paternal BMI is correlated to a higher risk of developing childhood obesity [26,27]. Interestingly, the effects of paternal BMI on the metabolic health of the offspring seem to be sexually dimorphic. In this regard, paternal obesity impairs intrauterine growth of male but not female offspring [28]. However, one of the main limitations of these epidemiological studies is the possibility that most of the observed phenomenon could be attributed to a shared obesogenic environment (i.e., an overabundance of high-caloric food in the household), rather than true transgenerational inheritance.

Previous studies have shown that maternal obesity induced by HFD in rats induces increased body weight gain in the F1 generation due to an increased daily caloric intake [29]. The observed weight gain agrees with our observations; however, we did not observe an increase in food intake. However, Oshio and colleagues (2019) using Wistar rats did not observe increased body weight in males sired by obese fathers [30]. This discrepancy could be attributed to the fact that their F0 generation was fed an HFD for a shorter period (91 vs. 126 days). In fact, their F0 _HFD_ did not present a significantly higher body mass compared to the control group. Similarly, Cesar and colleagues (2022) did not observe increased body weight in the progeny of obese males, even though their F0 _HFD_ displayed a significant increase in body weight [31]. Conversely, another report shows that paternal obesity potentiates weight gain induced by HFD consumption in F1 rats [32]. Unfortunately, this paper did not run an F1 group from obese fathers fed with a standard diet as a control. Finally, while there are some discrepancies regarding weight gain in F1 rats, our results agree with what has been observed by Fullston and colleagues in mice [12,15,16]. In addition, while F0 _HFD_ developed hyperglycemia and altered glucose homeostasis, individuals from F1 _HFD_ did not manifest such metabolic alterations, which agrees with previous reports on Wistar rats [30]. On the other hand, evidence from Sprague–Dawley rats is conflicting. One study reported that paternal HFD can induce pancreatic dysfunction and altered glucose homeostasis in female progeny, without increased body weight or adiposity [11]. While a more recent report did not find such metabolic alterations in neither male nor female F1 from HFD-fed parents [33]. In contrast, Fullston and colleagues have reported that paternal-obesity-induced hyperglycemia and insulin resistance in C57B/6 mice [15,16]. Thus, the transmission of these metabolic disturbances in rats might have incomplete penetrance.

Regarding reproductive parameters, while HFD caused increased body weight and adiposity in F0 males, we did not observe any significant alterations in seminiferous tubule morphology. This is in contrast to what has been reported in Wistar rats by other investigators; HFD ingestion significantly reduced testicular weight, tubular area, and the thickness of the germinal epithelium [30,34]. However, despite the seemingly normal testicular morphology, we observed a reduction in total sperm count and viability, compatible with the aforementioned studies. Therefore, our HFD treatment had an adverse effect on spermatogenesis, which is relevant considering that epigenetic changes in sperm have been linked to transgenerational inheritance of metabolic and reproductive disturbances [35].

In the case of F1 males, we did not observe significant alterations in testicular weight or morphology between groups. These findings agree with previous reports on mice [35,36] and rats [33]. In contrast, one report on Wistar rats indicated that while testicular weight was similar in F1 males, the germinal epithelium was thinner in F1 _HFD_ [30]. It is worth mentioning that while this report identified this morphological alteration, no further reproductive phenotype was observed. Despite the absence of any gross testicular alterations, we observed a significant reduction in viable sperm from the F1 _HFD_ group. Spermatic alterations have been reported in males sired by HFD-fed males [36,37]; however, what specific cellular event is being affected during spermatogenesis is unclear. Evidence shows that intergenerational reproductive anomalies induced by environmental toxins can be transmitted through alterations in Sertoli cell function [38]. Thus, we hypothesized that there could be alterations in Sertoli cell number with a concomitant decrease in spermatogonial proliferation. Nevertheless, there were no significant changes in WT1 or PCNA staining between groups. Furthermore, there were no changes in the total number of sperm cells; in addition, there was an absence of gross morphological alterations in the seminiferous tubules. Thus, indicating that the mechanisms implicated in the reduced sperm viability might involve affectations in interstitial tissues or in accessory reproductive organs. As a possible mechanism, we assessed plasma testosterone, which could reflect an impairment in Leydig cells. However, the observed difference did not reach statistical significance (*p* = 0.06). Thus, we cannot fully exclude reduced testosterone as a mechanism. One of the possible follow-ups to this work would be to assess changes in the expression levels of the enzymes responsible for its biosynthesis. Additionally, it has been observed that epididymal oxidative stress induced by HFD could affect sperm viability [39]. Furthermore, paternal obesity has been shown to reduce testosterone plasma concentration in F1 males [33], suggesting that there might be an impairment in Leydig cell function. Moreover, in mice, it has been shown that paternal HFD alters testicular metabolite content in their progeny [40], a phenomenon dubbed “inherited metabolic memory” of testicular tissue. Additionally, there are reports that indicate that endocrine disruptors induce prostate disease, even in the absence of testicular alterations [41]. Finally, increased oxidative stress along the reproductive system could be involved in the reduced sperm viability. Maternal obesity has been shown to disturb testicular oxidative stress homeostasis, along with a reduction in sperm quality [42].

There is evidence that maternal obesity can negatively affect the reproductive parameters of its female progeny. F1 from obese mothers have increased body weight, a precocious vaginal opening and increased estradiol concentration from postnatal day 1 to 60 [43]. Our results show that while paternal obesity induces increased body weight in F1 females, there are no differences on the day of the first vaginal opening, indicating that there are no alterations in the onset of puberty. To our knowledge, this is the first study to evaluate puberty onset and estrous cyclicity in female rats sired by obese males. While our model did not show any alterations in estrous cycle duration or rhythmicity, there is evidence that paternal exposure to fenvalerate can increase the duration of the estrous cycle in F1 females [44], confirming the notion that paternal metabolic status can impair the reproductive health of their female offspring. One possible mechanism for this phenomenon includes the changes in the ovarian secretion of steroid hormones [43,44]. Furthermore, we observed a reduction in the number of primordial follicles in F1 _HFD_ females, without any changes in the proportion of growing follicles; this is the first report to show such a phenomenon. There is prior evidence of maternal obesity impairing follicular growth [43] and paternal obesity inducing a reduction in the meiotic competency of oocytes [36]. Interestingly, this reduction in primordial follicles without changes in any other follicle category suggests that paternal obesity impairs follicular reserve, rather than follicular recruitment and growth. Primary ovarian insufficiency (POI) stems from a reduction in follicular reserve. While some risk factors for POI have been identified, the precise cause is often unknown; therefore, a possible paternal influence in the pathogenesis of POI is an intriguing hypothesis. There is previous evidence of a transgenerational transmission of reduced ovarian reserve due to maternal exposure to endocrine disruptors [45].

Until recently, research in the area of the DOHaD has been focused mainly on the maternal influence. However, multiple lines of evidence have accumulated in support of the role of paternal health around the conception period. While most of this research has focused on metabolic health of the offspring, this study, in addition to work from other groups [11,14,15], provides proof that paternal obesity can not only affect the metabolic health of rat progeny, but also reduce their reproductive potential. One of the main strengths of this study is the comparison of the intergenerational reproductive dysfunction in both sexes of the F1 generation. Additionally, it is one of the first studies to assess gametogenesis and gonad morphology in the progeny of obese males. We observed a reduction in viable sperm, even in the absence of an evident testicular affectation. On the other hand, we identified a significant decrease in primordial follicles in the ovaries of females sired by obese males. This indicates that the intergenerational inheritance of reproductive dysfunction might be sexually dimorphic.

## Figures and Tables

**Table 1:** Reproductive parameters of F0 males after 18 weeks of diet administration. Values represent mean ± SEM; * and ** scripts represent *p* ≤ 0.05 and ≤0.01, respectively. *n* = 12 and 6 for HFD and CTRL, respectively.

Condition	Live OffSpring Per Litter	Testis Weight (% of Body Mass)	Seminiferous Tubule Area (μm^2^)	Germinal Epithelium Thickness (μm)	Sperm Per mL (Millions)	% of Live Sperm	Plasma Testosterone (ng/mL)
Control	13.0 ± 1.3	0.416 ± 0.015	63,567 ± 1563	76.66 ± 1.16	30.64 ± 1.81	87 ± 3.59	37.21 ± 13.79
HFD	11.4 ± 1.2	0.378 ± 0.009 *	65,264 ± 1027	77.74 ± 0.86	20.64 ± 1.10 **	72 ± 3.55 **	17.25 ± 4.65

**Table 2:** Reproductive parameters of 18-week-old F1 males. Values represent mean ± SEM; ** script represents ≤ 0.01. *n* = 10 and 6 for F1 _HFD_ and F1 _CTRL_, respectively.

Condition	Testis Weight (% of Body Mass)	Seminiferous Tubule Area (μm^2^)	Sperm Per mL (millions)	% Of Live Sperm
F1 _CTR_	0.48 ± 0.02	6187 ± 3427	24.18 ± 2.63	92.8 ± 2.0
F1 _HFD_	0.47 ± 0.02	52,675 ± 2352	24.21 ± 1.34	75.2 ± 4.1 **

## Data Availability

The data presented in this study are available upon reasonable request from the corresponding author. Data is not publicly available due to privacy.
